# The timeline and risk factors of clinical progression of COVID-19 in Shenzhen, China

**DOI:** 10.1186/s12967-020-02423-8

**Published:** 2020-07-03

**Authors:** Fang Wang, Mengyuan Qu, Xuan Zhou, Kai Zhao, Changxiang Lai, Qiyuan Tang, Wenjie Xian, Ruikun Chen, Xuan Li, Zhiyu Li, Qing He, Lei Liu

**Affiliations:** 1 Department of Hepatology, Shenzhen Third People’s Hospital, National Clinical Research Center for Infectious Disease, The Second Affiliated Hospital, School of Medicine, Southern University of Science and Technology, 29 Bulan Road, Shenzhen, 518112 Guangdong China; 2 Institute of Reproductive Health, Tongji Medical College, Huazhong University of Science and Technology, Wuhan, China

**Keywords:** COVID-19, Pneumonia, Clinical progression, Risk factor, Retrospective analysis

## Abstract

**Background:**

The novel coronavirus disease 2019 (COVID-19) broke out globally. Early prediction of the clinical progression was essential but still unclear. We aimed to evaluate the timeline of COVID-19 development and analyze risk factors of disease progression.

**Methods:**

In this retrospective study, we included 333 patients with laboratory-confirmed COVID-19 infection hospitalized in the Third People’s Hospital of Shenzhen from 10 January to 10 February 2020. Epidemiological feature, clinical records, laboratory and radiology manifestations were collected and analyzed. 323 patients with mild-moderate symptoms on admission were observed to determine whether they exacerbated to severe-critically ill conditions (progressive group) or not (stable group). We used logistic regression to identify the risk factors associated with clinical progression.

**Results:**

Of all the 333 patients, 70 (21.0%) patients progressed into severe-critically ill conditions during hospitalization and assigned to the progressive group, 253 (76.0%) patients belonged to the stable group, another 10 patients were severe before admission. we found that the clinical features of aged over 40 (3.80 [1.72, 8.52]), males (2.21 [1.20, 4.07]), with comorbidities (1.78 [1.13, 2.81]) certain exposure history (0.38 [0.20, 0.71]), abnormal radiology manifestations (3.56 [1.13, 11.40]), low level of T lymphocytes (0.99 [0.997, 0.999]), high level of NLR (0.99 [0.97, 1.01]), IL-6 (1.05 [1.03, 1.07]) and CRP (1.67 [1.12, 2.47]) were the risk factors of disease progression by logistic regression.

**Conclusions:**

The potential risk factors of males, older age, with comorbidities, low T lymphocyte level and high level of NLR, CRP, IL-6 can help to predict clinical progression of COVID-19 at an early stage.

## Background

Since December 2019, a novel coronavirus disease (formerly known as 2019-nCoV and now renamed COVID-19) had rapidly spread throughout China, leading to a global outbreak and causing considerable public health concern [1, 2]. Until February 28th, 2020, the latest update from China’s National Health Commission reported there had been 78959 confirmed cases of the infection. Despite the lower case fatality rate, COVID-19 has so far resulted in more deaths (2791) than severe acute respiratory syndrome (SARS) and middle east respiratory syndrome (MERS) combined (1632) [3].

Despite the increasing confirmed cases updated daily, the clinical investigation of affected patients was limited. In an early study in Wuhan of 138 hospitalized patients, the mortality was 4.3% and 26% of patients received intensive care unit (ICU) care [4]. However, another research from Zhejiang province reported that of the 62 patients studied, the symptoms were relatively mild compared with Wuhan’s situation, only one was admitted to ICU, and no patients died during the study [5]. The significant contrast between those two made us want to explore further. Conclusions drawn from Wuhan alone might be biased and could not be representative of overall conditions due to its overwhelmingly rapid transmission and limited medical resources at the very beginning of the outbreak [6]. Thus, infected cases from regions outside Hubei can better inform the disease’s epidemiological and clinical characteristics.

Shenzhen, located near Hong Kong, served as a Special Economic Zone in southern China, has a large population density and high mobility, and therefore faces a comparatively high epidemics danger and transmission risk. The third people’s Hospital of Shenzhen is the only designated hospital which is authorized to admit all patients confirmed with COVID-19 in Shenzhen. In this study, we aimed to retrospectively describe the clinical features and laboratory findings of COVID-19 and also focused on searching for possible risk factors for clinical progression of severe patients in Shenzhen, and hopefully, providing valuable experience of patient management and stratification for other metropolises overseas.

## Materials and methods

### Study design and participants

It is a retrospective, single-center case series of the 333 hospitalized patients diagnosed with COVID-19 in the third people’s hospital of Shenzhen. We recruited all the confirmed patients whose admission date was from January 10, 2020 to February 10, 2020. All 333 COVID-19 patients were classified as mild, moderate, severe or critically ill category at admission. The diagnostic standard and classifying criteria of COVID-19 were based on the interim guidance from the WHO [7].

Two cohorts were generated in our research: 323 patients with mild-moderate symptoms on admission were observed for at least 18 days to determine whether they exacerbated to severe-critically ill conditions (progressive group) or not (stable group). In addition, we analyze the other 10 patients left who were severe-critically ill from the beginning of admission separately.

### Laboratory confirmation and data collection

Suspected cases were confirmed by positive real-time PCR assay for SARS-CoV-2. Pharyngeal swab specimens were collected on admission and may test several times for the COVID-19 confirmation. Other laboratory assessments included the whole blood count, electrolytes, coagulation test, liver and renal function, myocardial zymogram, C-reactive protein (CRP), procalcitonin, lactate dehydrogenase (LDH), Erythrocyte sedimentation rate (ESR), arterial O2/CO2 pressure and the like. Moreover, a typical chest computed tomography (CT) include multifocal bilateral ground-glass opacity with patchy consolidation. Massive consolidation with small pleural effusions and even “white lung” can be seen in severe-critically ill COVID-19 pneumonia [8]. The blood samples and CT scan were acquired on admission.

We extracted the medical records of the patients with COVID-19 and collected all the detailed data upon admission, including the basic information, epidemiological feature, clinical characteristic, laboratory finding as well as chest CT imaging.

### Statistical analysis

Continuous variables were described as the medians and interquartile ranges (IQR). Categorical variables were summarized as the frequencies and percentages in each category. Mann–Whitney test were applied to continuous variables, and Chi square test or Fisher’s exact test were used for categorical variables. Univariate and multivariate logistic regression analysis were adopted to identify risk factors of disease progression, and the Mann–Whitney test was used. For comparisons, a two-sided α of less than 0.05 was considered statistically significant. Statistical analyses were conducted with SPSS software version 23.0.

## Results

### The epidemic trend and outline of the COVID-19

**Fig. 1:**
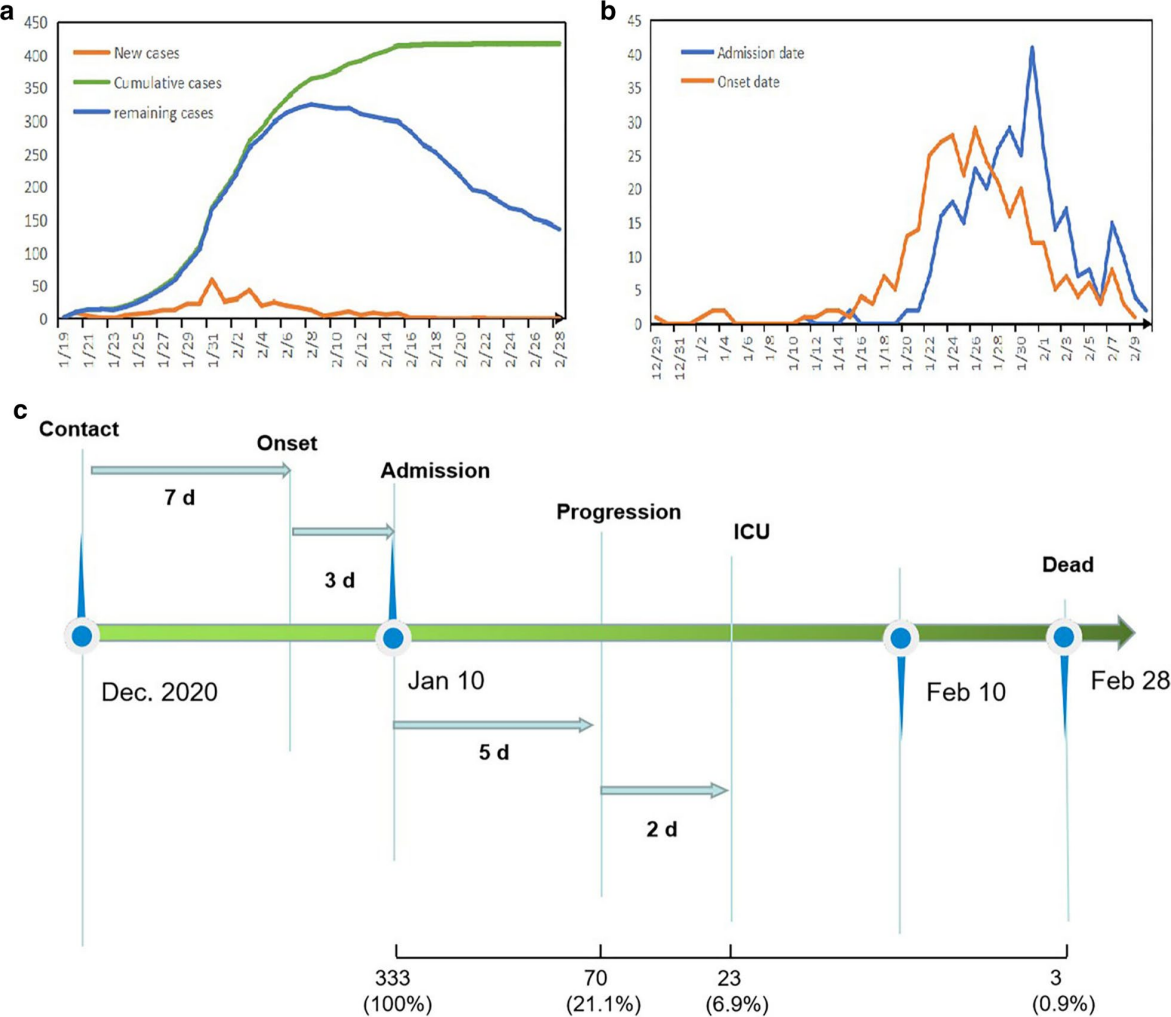
The epidemic trend and timeline in a COVID-19-designated hospital. **a** The outbreak of COVID-19 in Shenzhen according to official data from Jan. 10 to Feb. 28. **b** The admission date and onset date in the designated hospital. **c** The timeline of COVID-19 cases in the first month of admission

Located in the south of China with a population of 13.0 million, Shenzhen reported its first confirmed case on January, 19th 2020. Up until February 19th 2020, there were totally 417 cases confirmed according to the official reports. The epidemical trends of new cases, cumulative cases and remaining cases were shown in the Fig. 1a, newly confirmed cases per day reached the peak in around 12 days after first case report and the remaining cases started to decrease after about 20 days. Since February 18th, there were barely new cases added and the situation turned better.

To explore the timeline and disease progression of COVID-19, we focus on the 333 confirmed cases in the first month (admission date from Jan 10th to Feb 10th) (Fig. 1b). Most of the patients were admitted to hospital within 4 days after the disease onset, the median interval from disease onset to admission was 3 days (range 1–5 days) (Fig. 1c). In the 254 confirmed cases who had clear and credible information of exposure contacts to calculate the incubation period, the median of incubation period was 7 days (range 4–12 days). During the hospitalization, 70 (21.1%) mild-moderate cases progressed to severe condition in the median 5 days (range 2–8 days), 23 (6.9%) cases were admitted to ICU in median 2 days (range 1–4 days) after progression and unfortunately 3 (0.9%) patients died by the end of Feb 28th (Fig. 1c).

### The baseline clinical characteristics of disease progression

**Fig. 2:**
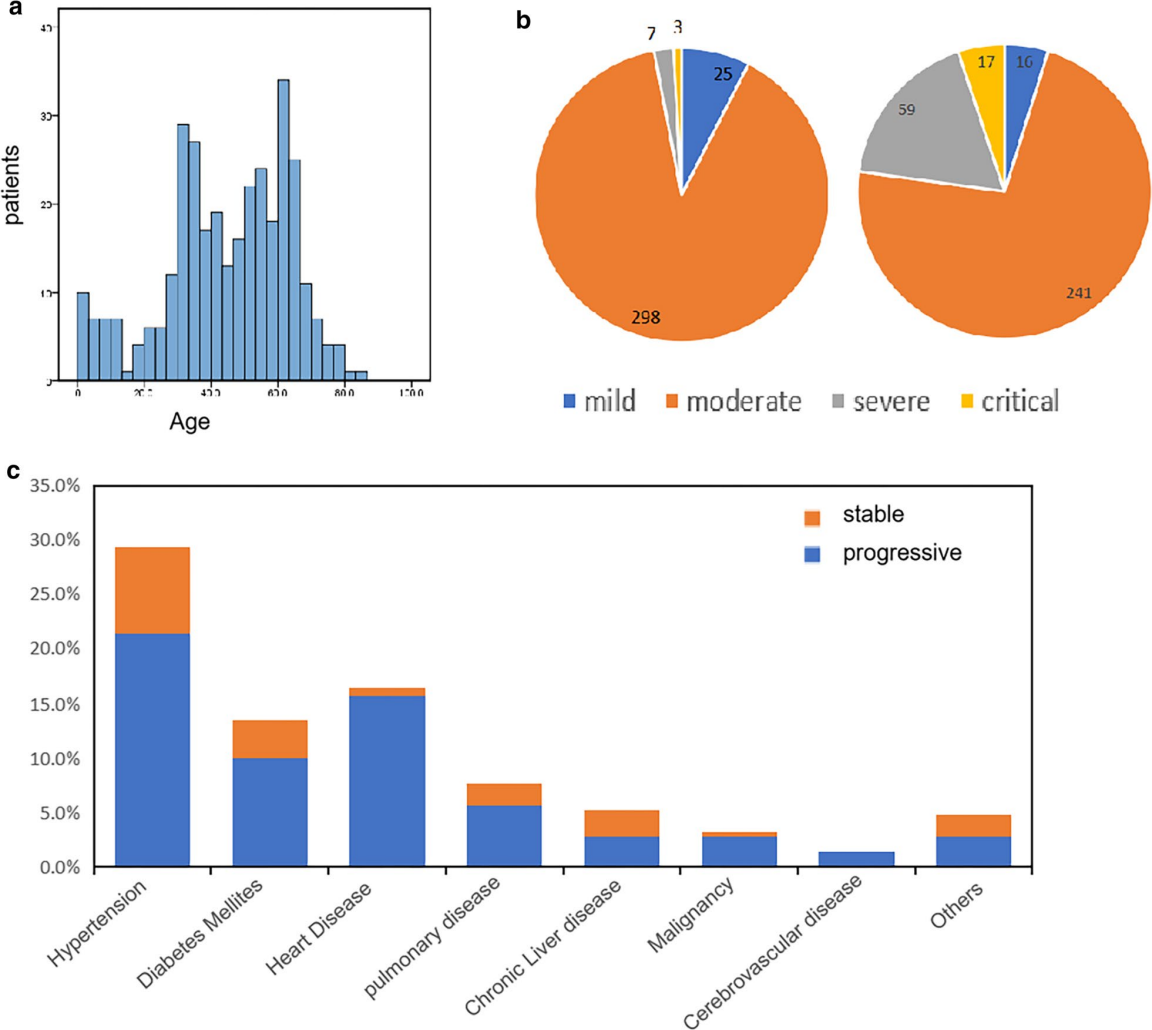
The distribution of age, symptom and baseline clinical characteristics. **a** The distribution of age in the COVID-19 patients; **b** The proportion of Clinical Severity of Confirmed COVID-19 Pneumonia on the admission (Left) and progressed period (Right). **c** The proportion in the progressed and stable patients

The median age of all the 323 patients was 46 years (IQR, 33–59; range, 8 months to 86 years), the age range and proportions were shown in the Fig. 2a. A total of 333 patients were classified according to the criteria defined above. The proportion of patients with mild, moderate, severe and critical on admission were 7.5% (25/333), 89.5% (298/333), 2.1% (7/333), and 0.9% (3/333), respectively. The spectrum of severity of diseases changed as disease progressed, 70 mild-moderate cases progressed to severe condition (progressive group), while 253 patients did not (stable group), and another 10 patients were severe from the beginning of admission (Fig. 2b).

Patients who later progressed to severe condition were more likely to have underlying comorbidities compared with the stable group (42.8% vs 16.6%, P < 0.05). Of all, hypertension was the most common disease (35, 10.8%), followed by diabetes (5.0%), heart diseases (4.0%), pulmonary disease (2.8%), liver diseases (2.5%), malignancy (0.9%), cerebrovascular disease (0.3%) and other conditions (2.1%) (Fig. 2c).

### The clinical characteristics of the progressive and stable groups

As shown in Table 1, compared with the stable group, the progressive group was significantly older (*P *< 0.001), there were no one under 18 years and patients over 65 years made up an evidently larger proportion (21.4%) in this group. More than half of all patients (169, 52.3%) were females, however, apparently more men (64.3%) ended up in progressive situation. Of all, 173 (53.6%) patients had an exposure history related to Wuhan and 90 (27.9%) cases were connected with other cities in Hubei province except Wuhan. Around 167 (51.7%) patients lived in Shenzhen but had outside contacts with confirmed or suspected infections or experienced a short term trip outside, whereas only 11 (3.4%) patients claimed no obvious exposure history. None of them were hospital-related transmission.

**Table 1:** Clinic characteristics and outlines of 323 patients infected with COVID-19 on admission

Characteristics	Total (N = 323)	Progressive (N = 70)	Stable (N = 253)	*P* value
Median age (years)	46.0 (33.0–59.0)	59.5 (49.0–64.0)	41.0 (32.0–56.0)	< 0.001
Age groups (years):				< 0.001
≤ 18	35 (10.8%)	0 (0%)	35 (13.9%)	
19–40	106 (32.8%)	9 (12.9%)	97 (38.3%)	
41–65	150 (32.5%)	46 (65.7%)	104 (41.1%)	
> 65	32 (9.9%)	15 (21.4%)	17 (6.7%)	
Sex:				0.002
Male	154 (47.7%)	45 (64.3%)	109 (43.1%)	
Female	169 (52.3%)	25 (35.7%)	144 (56.9%)	
Comorbidities	72 (22.3%)	30 (42.9%)	42 (16.6%)	< 0.001
Incubation period	7 (4,12)	7 (3–12)	7 (4.0–11.5)	0.994
From Onset to admission(days)	3 (1–5)	4 (2–6)	3 (1–5)	0.006
Hospital stay (days)	18 (14–22)	21 (18.5–23)	17.5 (14.0–21.0)	0.003
Exposure history:				
Living in Shenzhen with outside contact	167 (51.7%)	24 (34.3%)	143 (56.5%)	< 0.001
From outside to Shenzhen	127 (39.3%)	33 (47.1%)	94 (37.2%)	0.130
No obvious exposure	11 (3.4%)	5 (7.1%)	6 (2.4%)	0.051
Related to Wuhan	173 (53.6%)	40 (57.1%)	133 (52.6%)	0.490
Signs and symptoms				
Respiratory rate > 24 breaths per min	5 (1.5%)	3 (4.3%)	2 (0.8%)	0.036
Heart rate >100 per m in	62 (19.2%)	17 (24.3%)	45 (17.8)	0.222
Systolic pressure (mm Hg)	125.0 (115.0–136.0)	132.0 (123.5–140.5)	122 (112.0–133.0)	< 0.001
Temperature on admission	37.0 (36.6–37.5)	37.6 (36.9–38.0)	36.8 (36.6–37.3)	< 0.001
Fever	248 (76.8%)	61 (87.1%)	187 (73.9%)	0.154
Cough	160 (49.5%)	48 (68.6%)	112 (44.3%)	< 0.001
Expectoration	74 (22.9%	30 (42.9%)	44 (17.4%)	< 0.001
Chest tightness	16 (5.0%)	9 (12.9%)	7 (2.8%)	< 0.001
Dyspnea	9 (2.8%)	5 (7.1%)	4 (1.6%)	0.012
Myalgia or fatigue	69 (21.4%)	26 (37.1%)	43 (17.0%	< 0.001
Diarrhea	24 (7.4%)	7 (10.0%)	17 (6.7%)	0.354
Headache	26 (8.0%)	10 (14.3%)	16 (6.3%)	0.030
Anorexia	40 (12.4%)	15 (21.4%)	25 (9.9%)	0.009
Asymptomatic but nucleic acid positive	24 (7.4%)	2 (2.9%)	22 (8.7%)	0.100
Radiology manifestation				< 0.001
Unilateral involved	35 (10.8%)	3 (4.3%)	32 (12.6%)	
Bilateral involved	255 (79.0%)	67 (95.7%)	188 (74.3%)	
Normal	33 (10.2%)	0	33 (13.1%)	

Data are Median (IQR) or n (%), *P* value are calculated by χ^2^ test or Mann–Whitney U test

The most prevalent symptom was fever before admission (248,76.8%) and it was almost comparable between two groups (P = 0.154). Nearly half of patients were presented with pneumonia symptoms and systemic manifestations, including cough (49.5%), expectoration (22.9%), fatigue or myalgia (21.4%), anorexia (12.4%), dizziness (8.0%), chest tightness (5.0%), dyspnea (2.8%), and all of those symptoms were significantly more common and frequent in the progressive group. Notably, diarrhea and abdominal discomfort occurred in 7.4% of the patients and were slightly different in the progressive and stable cohorts (10.0% vs 6.7%). As for the vital signs, the progressive group tended to have significantly higher temperature and systolic blood pressure, and prone to tachypnea and low oxygenation index compared to the stable one. Interestingly, 24 patients were asymptomatic on admission but still timely hospitalized due to an exposure history and a laboratory-confirmed positive nucleic acid result of COVID-19 virus.

All patients underwent chest CT on admission, 255 (79.0%) patients presented bilateral pneumonia and 35 (10.8%) patients presented unilateral involved, while 33 (10.2%) patients showed almost no abnormalities. The progressive group displayed more lobes and segments involved, higher proportion of multiple ground-glass opacities, yet all 33 normal CT appeared only in the stable group.

### The laboratory parameters of the progressive and stable patients

As shown in Table 2, there were numerous differences in laboratory findings between the two groups. On admission, the progressive group presented slightly higher white blood cells and neutrophils (P = 0.026) than the stable one. However, the counts of Lymphocytes, T lymphocytes, CD4+ cell, CD8+ cell and platelets were significantly lower in the progressive patients, resulting in comparatively high level of Neutrophil-to-Lymphocyte Ratio (NLR). Generally, the baseline parameters representing the function of liver (alanine aminotransferase, aspartate aminotransferase, gamma glutamyl transferase), kidney (Creatinine Cr, blood urea nitrogen BUN) and myocardial zymogram (Troponin T, LDH) were distinctly elevated in the progressive group, indicating the potential organ dysfunction at the beginning. The blood levels of sodium, potassium and PO_2_, PCO_2_, oxygenation index were statistically lower in progressive patients, while elevated level of the infection-related indexes, i.e. ESR, CRP, procalcitonin, interleukin-6 (IL-6) were significantly more prevalent in this group on admission, as with the D-Dimer level. The preliminary results of blood test had already altered visibly in the progressive patients at early stage.

**Table 2:** Laboratory examination between the progressive and Stable patients

	Normal Range	Total (N = 323)	Progressive (N = 70)	Stable (N = 253)	*P* value
Blood cell count and lymphocyte classification					
White blood Cell (× 10^9^/L)	3.5–9.5	4.57 (3.55–5.65)	4.39 (3.62–5.72)	4.60 (3.57–5.63)	0.672
Neutrophils (× 10^9^/L)	1.8–6.3	2.56 (1.86–3.45)	2.84 (2.13–4.19)	2.52 (1.79–3.38)	0.026
Lymphocytes (× 10^9^/L)	1.1–3.2	1.27 (0.99–1.73)	1.02 (0.84–1.23)	1.38 (1.07–1.85)	< 0.001
NLR		1.90 (1.28–2.88)	2.72 (1.87–4.37)	1.72 (1.19–2.53)	< 0.001
Hemoglobin (g/L)	115–150	136.0 (127.0–146.0)	138.0 (128.5–148.5)	136.0 (127.0–146.0)	0.223
Platelets (10^9^/L)	125–350	180.0 (143.0–224.0)	147.0 (122.5–181.0)	190.5 (154.0–238.0)	< 0.001
T lymphocyte (N)	770–2041	980.0 (650.0–1317)	529.0 (387.0–712.5)	1071 (772.5–1399)	< 0.001
CD4 cell (N)	500–1500	525.0 (361.0–714.0)	302.0 (204.5–383.0)	596.5 (452.5–757.0)	< 0.001
CD8 cell (N)		356.0 (224.5–515.0)	201.0 (134.5–294.0)	402.5 (273.0–546.5)	< 0.001
CD4/CD8	1.5–2.5	1.49 (1.09–1.96)	1.61 (1.02–1.94)	1.48 (1.12–1.96)	0.907
Blood biochemistry					
Total bilirubin (uM)	1.7–21	9.80 (7.60–14.6)	10.4 (8.10–16.0)	9.55 (7.45–14.35)	0.303
ALT (U/L)	< 45	20.0 (15.0–30.1)	26.0 (19.0–38.3)	19.0 (13.0–27.0)	< 0.001
AST (U/L)	< 45	26.0 (21.0–35.0)	30.0 (23.5, 42.2)	25.0 (20.0–33.0)	< 0.001
GGT (U/L)	< 49	24.0 (15.3–39.0)	38.0 (23.0–62.0)	21.3 (14.5–32.0)	< 0.001
ALP (U/L)	35–100	60.0 (50.0–78.0)	59.0 (48.9–72.5)	60.5 (51.0–82.0)	0.101
Potassium (mM)	3.5–5.5	3.89 (3.62–4.15)	3.80 (3.53–4.09)	3.90 (3.64–4.17)	0.031
Sodium (mM)	135–145	138.2 (136.5–139.7)	136.2 (134.7–139.1)	138.4 (137.1–139.8)	< 0.001
BUN (mM)	2.6–7.5	3.92 (3.20–4.92)	4.78 (3.66–5.84)	3.80 (3.13–4.67)	< 0.001
Cr (uM)	41–73	62.0 (49.9–75.0)	71.0 (61.8–94.0)	58.0 (48.0–73.0)	< 0.001
eGFR (ml/min)	90–250	108.6 (96.2–119.7)	94.6 (77.0–105.6)	111.2 (98.0–121.2)	< 0.001
Troponin T (ug/L)	< 0.012	0.012 (0,006–0.012)	0.012 (0.012–0.013)	0.012 (0.006–0.012)	< 0.001
Creatine kinase	18.0–198.0	69.5 (50.0–96.0)	82.5 (56.0–126.0)	67.0 (48.5–90.5)	0.099
CK-MB (ng/mL)	0–2.37	0.54 (0.22–1.06)	0.62 (0.22–1.19)	0.55 (0.22–1.07)	0.426
LDH (U/L)	153	218.0 (174.0–379.0)	283.0 (199.0–577.0)	207.0 (166.0–323.0)	< 0.001
Infection-related parameters					
ESR (s)	0–20	30.0 (15.0–49.0)	34.5 (26.0–51.0)	23.5 (13.0–43.0)	< 0.001
CRP (mg/L)	< 8	9.9 (3.84–26.2)	26.64 (10.4–48.6)	7.25 (2.80–19.07)	< 0.001
PCT (ng/mL)	< 0.1	0.04 (0.03–0.06)	0.06 (0.05–0.09)	0.03 (0.02–0.05)	<0.001
IL-6 (pg/mL)	< 7	10.6 (4.12–19.7)	22.8 (13.1–32.1)	7.77 (3.61–14.9)	< 0.001
Oxygenation index (mmHg)	400–500	420.0 (360.5–477.0)	355.5 (296.0–401.0)	439.0 (378.5–495.0)	< 0.001
PaO_2_ (mmHg)	75–110	92.3 (79.2–106.0)	75.8 (69.8–85.9)	97.1 (84.4–108.0)	< 0.001
PCO_2_ (mmHg)	35–45	39.0 (36.1–41.4)	37.2 (33.3–39.0)	39.6 (37.0–41.9)	< 0.001
Coagulation function					
PT (s)	11–15.1	11.8 (11.3–12.4)	12.0 (11.3–12.6)	11.8 (11.2–12.3)	0.088
APTT (s)	28–43.5	35.3 (32.5–38.5)	36.7 (34.6–40.2)	34.7 (31.8–38.3)	0.002
D-Dimer (s)	0–0.5	0.36 (0.26–0.53)	0.53 (0.35–0.64)	0.34 (0.25–0.50)	< 0.001

Data are Median (IQR), P value comparing Progressive and Stable group are calculated by Mann–Whitney U test. NLR:Neutrophil-to-Lymphocyte Ratio; ALT, alanine transaminase; AST, aspartate transaminase; GGT, gamma glutamyl transferase; ALP, alkaline phosphatase; CK-MB, creatine kinase-MB; LDH, lactate dehydrogenase; BUN, blood urea nitrogen; Cr, Creatinine; eGFR, estimated Glomerular filtration rate; ESR, Erythrocyte sedimentation rate; CRP, C-reactive protein; PCT, procalcitonin; IL-6, Interleukin 6; PT, prothrombin time; APTT, activated partia l thromboplastin time

### Treatments and outcomes of all 333 patients

As shown in Table 3, all of the 333 patients, most patients (71.7%) had oxygen therapy and all patients received antiviral treatment. For severe cases, there was a significantly higher proportion of patients used antibiotics (60.8%), corticosteroid and gamma globulin (both over 75%) for treatment compared with the non-severe one. The most frequently used antibiotics were cephalosporin and quinolones. The mainly corticosteroid administrated was Methylprednisolone, the dosage of which was 1–2 mg/kg/day, maximum used shall be less than 3–5 day. No opportunistic infection was found. All the severe patients had oxygen support. In addition, 23 patients were admitted to intensive care unit, 11 of them had to use the invasive mechanical ventilation and 5 patients switched to extracorporeal membrane oxygenation. The most common complication was acute respiratory distress syndrome (ARDS) which happened to 13 severe patients. Other included acute cardiac injury, acute renal injury, septic shock and multiple organ failure which led to death cases. All 3 death cases were males and over 60 years old, one coexisting with hypertension and another with chronic obstructive pulmonary disease. Two of them were severe-critically ill at admission. Still, more than 240 patients were recovered and discharged from the hospital by February 28th.

**Table 3:** The treatment between the progressive and stable patients

	Total (N = 333)	Mild-moderate (N = 254)	Severe-critical (N = 79)	*P* value
Treatment				
Antiviral therapy	333	254	79	
Antibiotic therapy	99 (29.7%)	51 (20.1%)	48 (60.8%)	< 0.001
Use of corticosteroid	90 (27.0%)	22 (8.67%)	68 (86.1%)	< 0.001
Use of gamma globulin	81 (24.3%)	18 (7.1%)	63 (79.7%)	< 0.001
Regulate intestinal flora	179 (53.8%)	123 (48.4%)	56 (70.1%)	< 0.001
Oxygen support	238 (71.5%)	160 (63.0%)	79 (100.0%)	
Nasal cannula	181 (54.4%)	157 (61.8%)	23 (29.1%)	< 0.001
Mask oxygen inhalation	7 (2.1%)	3 (1.2%)	4 (5.1%)	0.036
High-flow nasal cannula	10 (3.0%)	0	10 (12.7%)	
Non-invasive ventilation	24 (7.2%)	0	24 (30.4%)	
Invasive mechanical ventilation	13 (3.9%)	0	13 (16.5%)	
Invasive mechanical ventilation + ECOM	5 (1.5%)	0	5 (6.3%)	
Acute respiratory distress syndrome	13 (3.9%)	0	13 (16.5%)	

### The potential risk factors of disease progression

To predict the risk factors of disease progression based on the clinical features, we found that age, sex, history of exposure, comorbidities, radiology manifestation were significantly associated with the disease progression by the univariate logistic analysis. Furthermore, aged over 40 years, male sex, with comorbidities, a clear and certain exposure history and abnormal radiology manifestations were all risk factors for disease progression by the multivariate logistic analysis (Tables 4).

**Table 4:** Risk factors of basic information for progression by logistic regression

Variable	Univariate analysis		Multivariate analysis	
	OR (95% CI)	*P* value	OR (95% CI)	*P* value
Age (> 40 years vs ≤ 40)	7.39 (3.52, 15.53)	< 0.001	3.80 (1.72, 8.52)	0.001
Sex (male vs. female)	2.38 (1.37, 4.11)	0.002	2.21 (1.20, 4.07)	0.011
History of exposure (yes vs no)	0.249 (0.14, 0.44)	< 0.001	0.38 (0.20, 0.71)	0.002
Incubation period (days)	1.01 (0.95, 1.07)	0.723		
Comorbidities (yes vs no)	2.86 (1.87, 4.38)	< 0.001	1.78 (1.13, 2.81)	0.013
Radiology manifestation (yes vs no)	5.38 (1.89, 15.35)	0.002	3.56 (1.13, 11.40)	0.032
Symptoms (yes vs no)	1.02 (0.68, 1.53)	0.914		
Respiratory rate (> 24 breaths/min)	1.06 (0.92, 1.21)	0.480		

As shown in the Table 5 of laboratory parameters, the univariate logistic analysis suggested that the baseline levels of NLR, T lymphocyte, BUN, CRP, IL-6, ESR were significantly associated with the disease progression. However, the multivariate logistic analysis indicated that low T lymphocyte level and high levels of CRP, IL-6, NLR were risk factors for disease progression (Table 5).

**Table 5:** Risk factors of lab test for progression by logistic reprogression

Variable	Univariate analysis		Multivariate analysis	
	OR (95% CI)	*P* value	OR (95% CI)	*P* value
White blood cell (×10^9^/L)	0.97 (0.84,1.12)	0.655		
NLR	1.44 (1.23, 1.68)	< 0.001	0.99 (0.97, 1.01)	0.048
T lymphocyte	0.996 (0.994, 0.997)	< 0.001	0.99 (0.997, 0.999)	0.002
CD4/CD8	1.02 (0.68, 1.52)	0.939		
TBIL	1.02 (0.98, 1.06)	0.325		
ALP	0.99 (0.98, 1.00)	0.067		
Troponin T	0.92 (0.60, 1.51)	0.738		
CRP	1.04 (1.02, 1.05)	< 0.001	1.67 (1.12, 2.47)	0.012
PCT	0.90 (0.32, 2.51)	0.833		
IL-6	1.05 (1.03, 1.07)	< 0.001	1.03 (1.00, 1.05)	0.008
ESR	1.02 (1.00–1.03)	0.002	0.99 (0.97, 1.01)	0.529
D-Dimer	1.78 (1.02, 3.09)	0.052		
Ferritin	1.00 (1.000–1.001)	0.189		

NLR, Neutrophil-to-Lymphocyte Ratio; CRP, C-reactive protein; PCT, procalcitonin

### The diagnosis value and predictors of disease progression

**Fig. 3:**
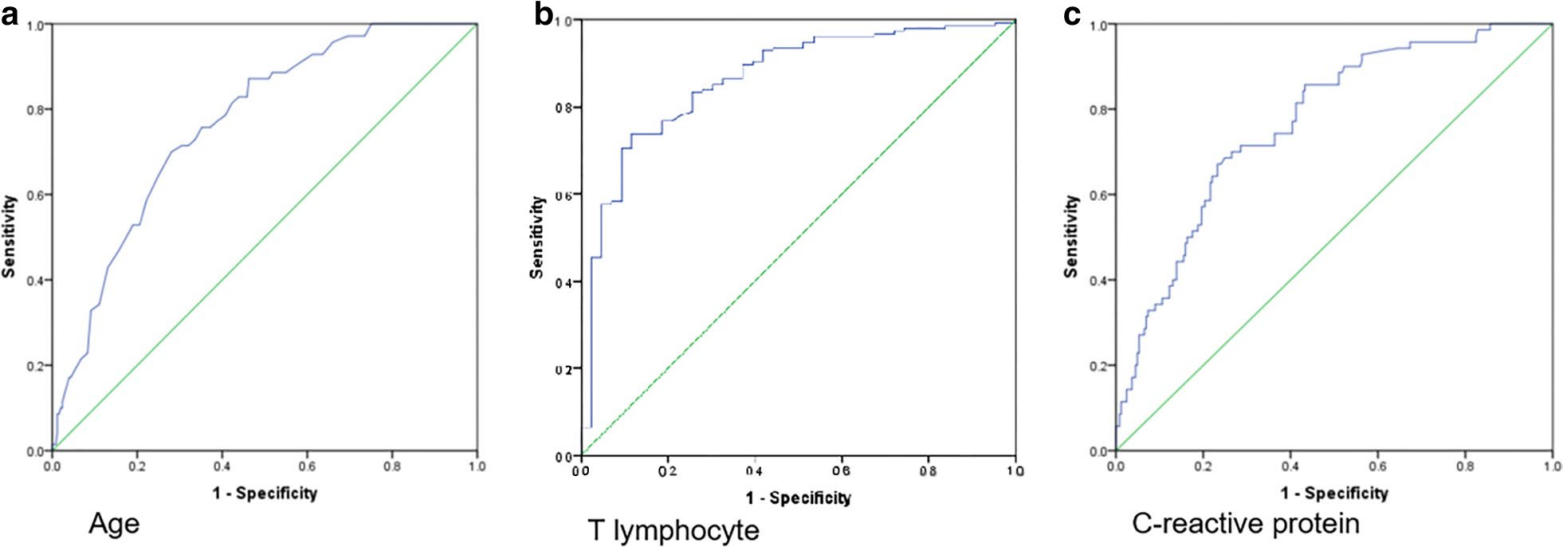
The ROC curve of age, T lymphocyte and CRP of the progressive and stable patients. **a** ROC curve of age; **b** ROC curve of T lymphocyte; **c** ROC curve of C-reactive protein

Furthermore, through the ROC curve test (Fig. 3), the best cut-off point of age (AUC = 0.767) was 53.5 years, with a specificity of 70% and a sensitivity of 28.1%. And the ROC curve of T lymphocyte (AUC = 0.865) suggested that the best cut-off point was 825/ul with a specificity of 88.4% and a sensitivity of 26.3%. The ROC curve of CRP (AUC = 0.0.768) suggested that the best cut-off point was 9.71 mg/ml with a specificity of 81.4% and a sensitivity of 41.2%.

**Fig. 4:**
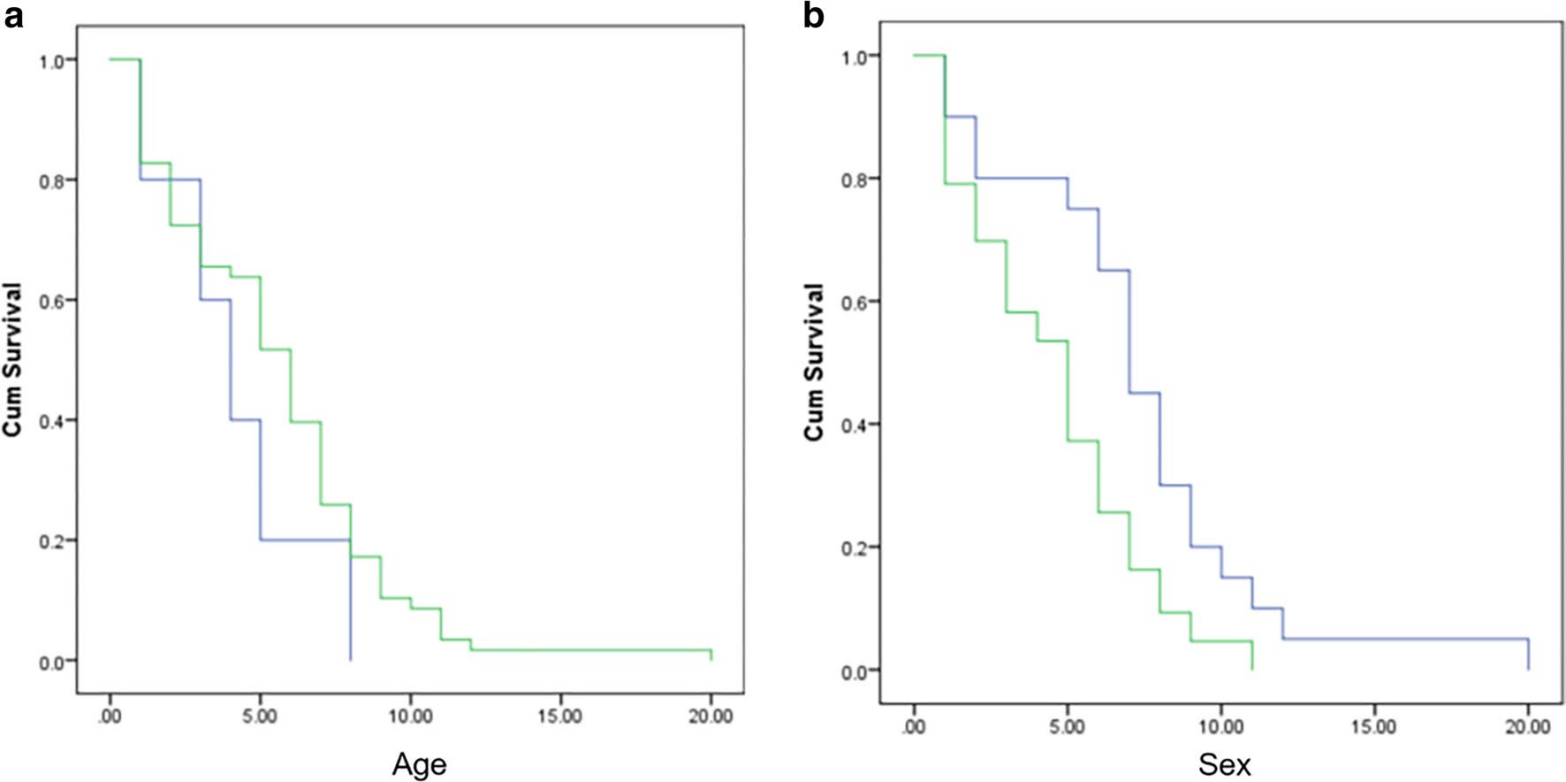
Comparison of the interval of disease progression between the progressive and stable patients. The interval of disease progression by age (**a**) and sex (**b**) by the Kaplan–Meier analysis

Compared with the stable group, the length of disease progressing time was significantly different according to the age and sex by the Kaplan–Meier analysis (Fig. 4). It can be inferred that the elderly and male patients were more likely to progress into severe-critically ill conditions.

## Discussion

Since the rapid person-to-person transmission of COVID-19 outbreak occurred in December 2019, the number of infected cases had risen exponentially. The world was on the brink of a pandemic [9, 10]. In our study, we retrospectively assessed the clinical characteristics and medical tests of 333 patients infected with COVID-19 in the only designated hospital in Shenzhen and tried to analyze and identify the baseline risk factors for clinical progression.

COVID-19 appeared to pose a particular threat to middle-aged and older adults, especially men, while it spared the underage group. None of the patients under 18 years (35 minors, 10.5%) deteriorated and all remained a mild-moderate condition. However, aged over 40 years had taken up a prominently high proportion (over 87%) of the progressive group, while patients under 40 years accounted for more than half of the stable group. Although men and women were infected in roughly equal numbers, the number of males in severe-critically ill condition was almost twice as compared to women (52 vs 27). Besides, all three death cases were male. Underling disease was another contributing factor. Nearly half of the severe cases were coexisting with at least one comorbidity. Patients with two or more comorbidities had a significantly elevated risk of exacerbation [11]. We also noticed that among all the COVID-19 infections, there were 4 patients infected with respiratory syncytial virus and 2 patients with influenza B virus concurrently, which did not result in aggravated condition but remained mild and moderate. In the early stage of the transmission, we found most infected cases were directly related to Wuhan or Hubei province and only 3.4% patients claimed unclear exposure history, which meant we can easily trace the source of virus transmission and isolate the suspected ones. Furthermore, patients who had an early onset and admitted to hospital before January 25th tended to be more severe and progressive, for 19 out of 48 patients (40%) developed to severe-critically ill conditions and two of them even died.

Until February 28th, more than 240 patients were recovered in our study, 62 (18.7%) of covid-19 infections were diagnosed as severe cases and 17 patients (5.1%) were critically ill, while the mortality rate was estimated 0.9%, much lower than Hubei region had reported [12]. Fever was the most universal symptom among all, but patients can be afebrile and respiratory symptoms were not presented in all cases. Unlike a common cold, sore throats and rhinorrhea or rhinobyon were relatively rare. Diarrhea might be underestimated since ACE2 was highly expressed in the small intestine which can be attacked by the virus [13]. Therefore, we should be cautious when a feverish patient with diarrhea saw a doctor. In terms of severe cases, high fever with systemic symptoms may be predictive for clinical progression [14]. All three death cases had multiple symptoms including fever, cough, dyspnea, fatigue, anorexia at admission and then developed into ARDS. In addition, a small proportion were asymptomatic patients screened out from the nucleic acid test and close contacts. It can be contagious as well and may threaten the life of other cohabiting members, especially the elderly ones. Notably, among the 24 carriers, 12 patients aged under 18 years and showed only mild symptoms during hospitalization. 15 patients had already shown the radiologic feature of COVID-19 pneumonia in chest CT scan upon admission. Only 2 of them progressed to severe while 22 remained stable and mild, which indicated the importance of continuous nucleic acid tests and early abnormalities detection in CT imaging [8, 15, 16]. Diagnosis in the early phase and isolated for medical observation may be helpful to the whole community.

In terms of laboratory test results, lymphocytes, especially T lymphocytes were significantly reduced in severe cases, which indicates COVID-19 consumed immune cells and inhibits the cellular immune function. In addition, progressive patients tend to have higher baseline NLR, ESR, CRP, IL-6, D-dimer level, which may be related to inflammatory response and cytokine storm induced by virus invasion [17, 18]. Our results were also in line with other retrospective studies [19, 20]. Those inflammatory factors level at admission may help to identify and determine later clinical progression. A higher level suggested more significant risk to exacerbation. Nearly all the patients received antiviral treatment. Lopinavir/ritonavir were reported to have potentially therapeutic effects on SARS and widely applied [21]. Antibiotics were strictly controlled and only prescribed to patients with a highly suspected bacterial infection in our hospital. Corticosteroid and gamma globulin were typically used in severe cases to reduce lung inflammatory response.

The reason for the rapid expansion might be associated with the mild and atypical symptoms in the early stage of infected individuals. As there were no specific and effective antiviral therapies identified, our suggestion was to control the source of infection, as well as the use of facial mask for protection, isolation and early diagnosis. More importantly, early identification of risk factors associated with the clinical progression of COVID-19 should be paid prominent attention for better patient management and stratification.

The study is subjected to certain limitations. Firstly, as the epidemics has not ended yet and many patients are still hospitalized at the time of study submission, we are unable to estimate the overall proportion of clinical progression and case fatality rate. Secondly, due to the retrospective nature of the study, a systematic selection bias and residual confounding factors cannot be fully addressed and may lead to inaccurate conclusion. Thirdly, the clinical predictive value remains to be explored and a multi-center and follow-up study with a larger cohort is highly required.

## Conclusion

In this single-center case series of 333 hospitalized patients with confirmed COVID-19 in Shenzhen, China, we assessed and analyzed the clinical characteristics and potential predictors of disease progression and prognosis on admission and found the risk factors of males, older age, with comorbidities, low T lymphocyte level and high level of NLR, CRP, IL-6 can help to predict clinical progression of COVID-19 at an early stage.

## Data Availability

The data supporting the conclusions of this article are included within the article.
